# Identification of Turtle-Shell Growth Year Using Hyperspectral Imaging Combined with an Enhanced Spatial–Spectral Attention 3DCNN and a Transformer

**DOI:** 10.3390/molecules28176427

**Published:** 2023-09-04

**Authors:** Tingting Wang, Zhenyu Xu, Huiqiang Hu, Huaxing Xu, Yuping Zhao, Xiaobo Mao

**Affiliations:** 1 School of Electrical and Information Engineering, Zhengzhou University, Zhengzhou 450001, China; wangtingting_mail@163.com (T.W.); xzy991206@163.com (Z.X.); huhuiqiang_zzu@163.com (H.H.); 2 China Academy of Chinese Medical Sciences, Beijing 100700, China; zhaoyuping0316@hotmail.com; 3 Research Center for Intelligent Science and Engineering Technology of Traditional Chinese Medicine, Zhengzhou University, Zhengzhou 450001, China

**Keywords:** hyperspectral imaging, turtle shell, growth-year identification, spatial–spectral attention, 3D convolutional neural network, transformer

## Abstract

Turtle shell (*Chinemys reecesii*) is a prized traditional Chinese dietary therapy, and the growth year of turtle shell has a significant impact on its quality attributes. In this study, a hyperspectral imaging (HSI) technique combined with a proposed deep learning (DL) network algorithm was investigated for the objective determination of the growth year of turtle shells. The acquisition of hyperspectral images was carried out in the near-infrared range (948.72–2512.97 nm) from samples spanning five different growth years. To fully exploit the spatial and spectral information while reducing redundancy in hyperspectral data simultaneously, three modules were developed. First, the spectral–spatial attention (SSA) module was developed to better protect the spectral correlation among spectral bands and capture fine-grained spatial information of hyperspectral images. Second, the 3D convolutional neural network (CNN), more suitable for the extracted 3D feature map, was employed to facilitate the joint spatial–spectral feature representation. Thirdly, to overcome the constraints of convolution kernels as well as better capture long-range correlation between spectral bands, the transformer encoder (TE) module was further designed. These modules were harmoniously orchestrated, driven by the need to effectively leverage both spatial and spectral information within hyperspectral data. They collectively enhance the model’s capacity to extract joint spatial and spectral features to discern growth years accurately. Experimental studies demonstrated that the proposed model (named SSA–3DTE) achieved superior classification accuracy, with 98.94% on average for five-category classification, outperforming traditional machine learning methods using only spectral information and representative deep learning methods. Also, ablation experiments confirmed the effectiveness of each module to improve performance. The encouraging results of this study revealed the potentiality of HSI combined with the DL algorithm as an efficient and non-destructive method for the quality control of turtle shells.

## 1. Introduction

Turtle shells, also known as “Guijia” in Chinese, are the dorsal and ventral shells of the tortoise *Chinemys reevesii* (Gray), a member of the family Tortoiseidae, and have been used as medicine and dietary therapy in China for 1700 years, making them one of the most valuable traditional Chinese herbal medicines [1]. Meanwhile, herbalists believe that it can benefit kidneys, invigorate bone, regulate menstruation, and relieve metrorrhagia [2]. Regrettably, the drive to minimize expenses and maximize profits has led to the sale of subpar turtle shells at inflated prices, creating a mixture of qualities that cannot guarantee safety or effectiveness [3]. Among the factors that affect the efficacy of turtle shells, the most critical determinant is the content of active ingredients in turtle shells. The magnitude of active ingredients is influenced by a myriad of factors, encompassing variations in growth environments, although preeminent among these determinants is the growth years. As the growth years of turtle shells increase, the content of amino acids, zinc, manganese, and copper elements gradually increases, and the healing effect of turtle shells is better. Consequently, the growth year of turtle shells is an important basis for turtle-shell quality inspection.

Visual inspection is the traditional method used for detecting and identifying herbal medicine [4]. The inspector makes an empirical identification of the merits and approximate age of turtle shells based on the color and texture of the turtle shell’s surface and other characteristics, but it is either subjective in nature or requires extensive experience. With the advancement of chemical detection technology, there exist identification techniques using component detection to assess quality [5], such as chromatography (e.g., gas chromatography–mass spectrometry (GC–MS) [6,7], high-performance liquid chromatography (HPLC) [8]), the polymerase chain reaction (PCR) technique [9], and the polyacrylamide gel electrophoresis (SDS–PAGE) technique [10]. Despite their advantages, these sophisticated methods have drawbacks as they are invariably expensive, time-consuming, and require complicated sample pretreatment and highly skilled technicians. In addition, these methods are not repeatable as they are performed on representative samples that cannot guarantee the quality of the entire batch, leading to inaccurate quality assessment [11]. Accordingly, the existence of non-destructive, rapid, and efficient quality-control methods for turtle shells would be convenient to ensure their great commercial and nutritional values.

Recently, there has been growing interest in a nondestructive technology, referred to as hyperspectral imaging (HSI). This technique integrates spectroscopic and imaging methodologies into a unified system, allowing for the simultaneous capture of spatial and spectral information. By allowing for rapid, efficient, and non-invasive analysis, this technique permits the online monitoring of quality and safety control in various fields, presenting significant advantages. Based on such advantages, this technology, coupled with chemometrics, has also been utilized for the quality evaluation of herbal medicine [12,13,14,15,16].

For example, Wang et al. [17] applied random forest (RF) for harvesting period discrimination in *Dendrobium officinale* and obtained 94.44% accuracy. Ru et al. [18] extracted effective wavelengths from spectral data using the successive projection algorithm (SPA) and combined them with a textural variable; then, they used partial least-square–discriminant (PLS–DA) for classifying the geographical origins of Rhizoma Atractylodis Macrocephalae and achieved an accuracy of 93.2%. Han et al. [19] used the support vector machine (SVM) discriminant model based on characteristic bands established by principal component analysis (PCA) to classify glycyrrhiza seeds, and the accuracies of the training set and test set were more than 93%. Wang et al. [20] introduced a near-infrared hyperspectral wavelength selection method using one-way analysis of variance (ANOVA), which outperformed PCA and the genetic algorithm (GA) with an average accuracy of 95.25% on the test set after 10-fold cross-validation. Yao et al. [21] developed a marine predator’s algorithm–least-squares support vector machine (MPA–LSSVM) model to classify the grades of Panax notoginseng powder by analyzing its spectral data using a combination of competitive adaptive reweighted sampling (CARS) and PCA, achieving 96.67% and 95% accuracy rates for the training and test sets, respectively.

The presence of numerous redundant and highly correlated spectral data in hyperspectral images can lead to the Hughes phenomenon, which poses a challenge for classification tasks. For the abovementioned traditional machine learning (ML) algorithms [18,19,20,21], appropriate feature spectral selection is necessary, which has a considerable impact on classification performance. In practice, there are many feature selection/extraction methods that can be referenced [14,22], and usually, the optimal results are reported through a cumbersome trial-and-error process in the literature, which heavily relies on experts’ domain knowledge and results in inconvenience [23]. Recently, deep learning (DL), particularly the convolutional neural network (CNN), has emerged as a promising candidate for developing a general and automated approach due to its ability to represent complex data without extensive manual feature engineering [23].

In the last few years, DL has been increasingly applied in the hyperspectral-based quality control of herbal medicine and achieved appealing classification performance. For instance, Yan et al. [24] used visible/near-infrared (Vis/NIR) and near-infrared (NIR) HSI to identify *Radix glycyrrhizae* in four different geographical origins. This study constructed DL models (recurrent neural network (RNN) and CNN) from one-dimensional spectra, comparing them with logistic regression (LR) and SVM models. The results highlighted CNN’s proficiency with Vis/NIR spectra and RNN’s superiority with NIR spectra. Kabir et al. [25] employed HSI integrated with CNN to differentiate twelve Fritillaria varieties, achieving superior accuracy compared to PLS–DA and SVM models, with training and test set accuracies of 98.88% and 88.89%, respectively. Dong et al. [26] integrated two-dimensional–correlation spectroscopy (2D–COS) of NIR hyperspectral images with CNN to discriminate the origin of wolfberries. By utilizing the optimized fusion dataset, the CNN model showed exceptional results, with 100% accuracy in the calibration set and 97.71% in the prediction set. Mu et al. [27] presented a novel new hybrid convolutional neural network (new hybrid CNN) approach that utilizes the spectral–spatial joint information of HSI for categorizing four different origins of wolfberry. The findings indicate that the new hybrid CNN is more effective than the SVM that focuses only on spectral features.

Motivated by this success, the current study aims to explore the viability and effectiveness of utilizing the NIR hyperspectral reflectance imaging technique as a non-invasive, fast, and efficient method for turtle-shell growth-year identification. Importantly to accomplish this, we propose a model that combines the spectral–spatial attention mechanism, 3DCNN, and a self-attention-based transformer to effectively extract discriminative spectral–spatial joint information from the hyperspectral data. Several noteworthy contributions arise from our study, which can be summarized as follows:

- First, inspired by the study in [28], a spectral–spatial attention mechanism module is developed to selectively extract the low-level features of the neighborhood pixel blocks and reduce spectral redundant information in raw 3D HSI data. Generally, extracting only spectral data from a region of interest (ROI) as a one-dimension vector could lead to the loss of external spatial information, or spectral and image information of HSI data could be considered separately [29]. Also, this wealth of spatial and spectral information of 3D hyperspectral images unequally contributes to the final classification, particularly considering the correlation and redundancy within the spectral spectrum. To deal with the aforementioned challenges, the proposed model utilizes a sequential stacking of the spectral–spatial attention (SSA) module to refine the learned joint spectral–spatial features.
- Second, regarding the extracted 3D feature map, three-dimensional convolution and the transformer are employed to effectively capture the local and global information for optimizing the classification process. Joint spatial–spectral feature representation is facilitated by 3D convolution. Moreover, taking into account that HSI can be perceived as sequence data, the convolution operation is confined by its receptive field, hence it cannot maintain a balance between model performance and depth [30]. To overcome these limitations and enhance the efficacy of spectral–spatial feature extraction, a novel approach was adopted. This approach integrated a transformer encoder block with multi-head self-attention (MSA) mechanisms, called the TE block, which can effectively address the long-distance dependencies inherent in the spectral band information of the hyperspectral image data.

Validated on collected datasets, experimental studies demonstrate that the proposed method achieved superior classification accuracy compared with representative algorithms. In addition, ablation experiments were conducted and confirmed that the modules introduced could provide consistent performance gain.

The remaining content of this article is outlined as follows. Experimental studies and a discussion of results are presented in Section 2. Section 3 presents hyperspectral image data acquisition and the necessary preprocessing process. Section 4 illustrates the details of the proposed deep learning model, as well as the principles of each module. Finally, the conclusions are given in Section 5.

## 2. Results and Discussion

In this section, we first illustrated the spectral profile of turtle shells from different growth years, and then the hyperparameters that influence the performance of the proposed model were investigated and selected. After that, ablation experiments were conducted to investigate the influence of different modules on the proposed model. Finally, we proceeded to compare the performances of various models utilizing full spectral information, followed by an extended comparative analysis involving the proposed model and several deep learning architectures, thereby discussing the obtained comparative outcomes.

### 2.1. Spectral Profile

**Figure 1:**
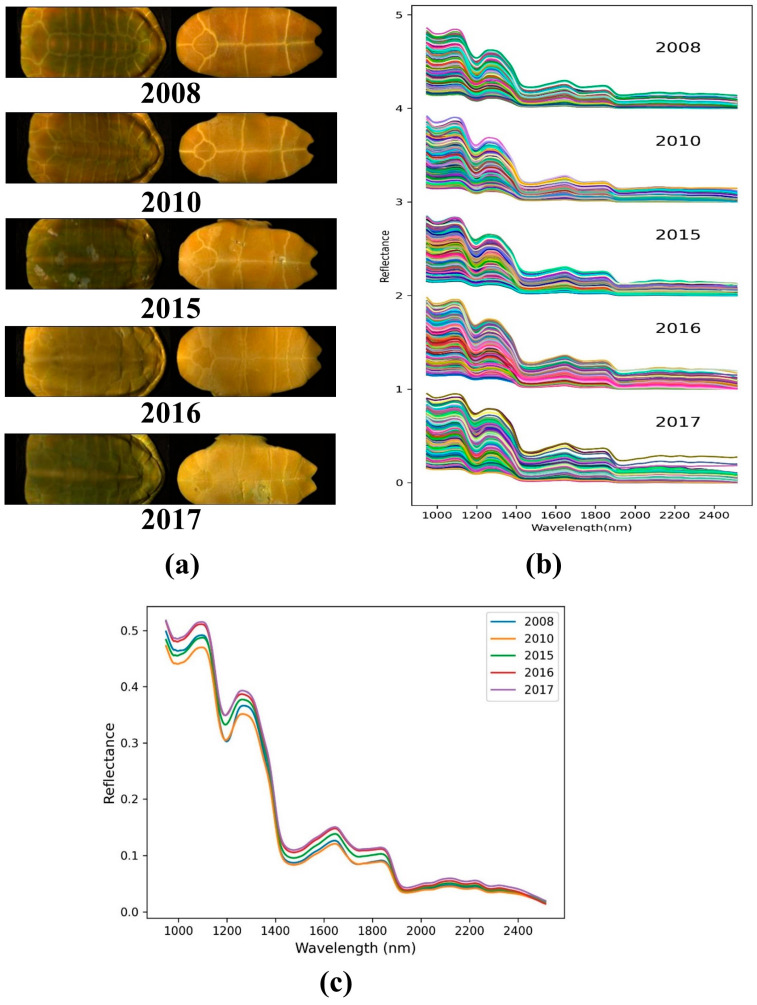
The spectral profiles of turtle shells from five different growth years: (**a**) Pseudo-RGB image of turtle shells, (**b**) Spectra of all samples in the whole dataset, with each sample’s spectrum exhibit distinct colors (Note that the vertical coordinate of each category is moved up by 1 successively), (**c**) Average spectral for each growth year.

Figure 1 presents the spectral profiles of collected turtle shells from five different growth years. By observing Figure 1a, we found the pseudo-RGB image of different turtle shells to be very similar, indicating that identifying the growth year of turtle shells through their morphology and texture using the naked eye is difficult. Regarding Figure 1b, it was evident that all the spectral curves follow similar trends, but the reflectance intensities vary mainly in the 950–2000 nm range, showing the unique spectral characteristics of each sample. By further inspecting the average spectral curves, as shown in Figure 1c, it was found that there are spectral peaks at approximately 1097 nm, 1268 nm, 1647 nm, and 1844 nm, and spectral valleys at approximately 988 nm, 1191 nm, 1465 nm, 1735 nm, and 1923 nm. Furthermore, the reflectance values of these spectral features varied distinctly across different growth years of turtle shells. Generally, the observed peaks and valleys in the reflectance spectra are attributed to the periodic stretching vibrations of the C-H, O-H, and N-H bonds present in the proteins and amino acids [13], which form the fundamental chemical bonds of organic compounds. Therefore, the dissimilarity in the spectral features indicates that HSI has the capability to discriminate between different growth years of turtle shells.

### 2.2. Parameter Analysis

In the parameter analysis, an in-depth investigation was conducted into the effect of several crucial parameters that impact the training process and classification performance. These parameters include the number of principal components, learning rate, number of 3D convolution kernels, and number of heads in the MSA. Other essential parameters such as optimizer, batch size, and number of epochs were set as described in Section 4.5.

**Figure 2:**
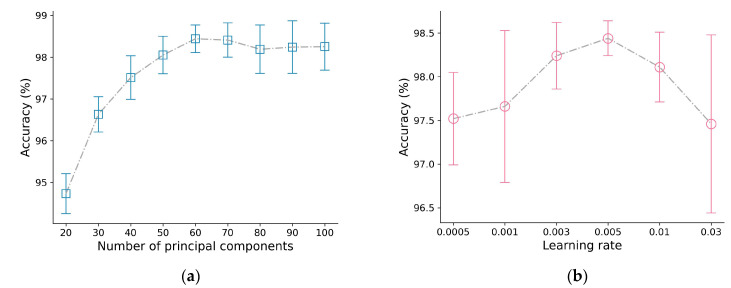

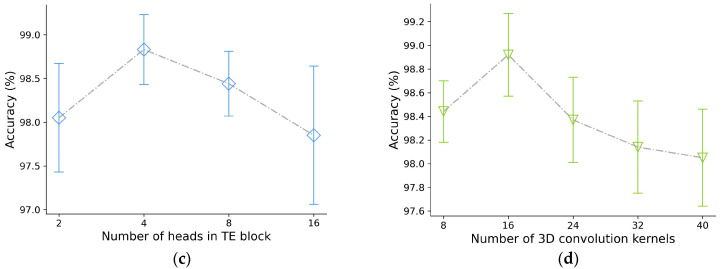
Performance of SSA–3DTE model under different parameters: (**a**) number of principal components, (**b**) learning rate, (**c**) number of heads in TE block and (**d**) number of 3D convolution kernels.

We ran each experiment five times, and the experimental comparison results are shown in Figure 2.

(1) Principal component analysis: PCA was utilized to process the HSI data in order to mitigate the computational burden and spectral dimensionality. Here, the principal component numbers were evaluated as 20, 30, 40, 50, 60, 70, 80, 90, and 100. It can be seen from Figure 2a that the principal component numbers have an impact on the classification performance. Among them, the worst classification accuracy is 94.73% when the number of principal components is 20, and the highest is with 60 components. The main reason is that regarding the principal component number, if the setting is too small, most of the valid features will be rejected, and if the setting is too large, it may contain some redundant spectral information, also with an increased computational burden. Also, the model with 60 principal components maintains a smaller variance, which means that it obtains a relatively stable performance. For the subsequent trials, the principal component numbers are set to 60.
(2) Learning rate: To ensure effective training, selecting an appropriate learning rate is essential as it greatly affects the gradient descent rate of the model and influences the convergence performance and speed of the model. In this study, an analysis of various learning rates was conducted, including 0.0005, 0.001, 0.003, 0.005, 0.01, and 0.03. Figure 2b shows that an appropriate increase in the learning rate has a positive effect on the model performance, and the effect reaches an optimal value for accuracy with a learning rate of 0.005, but a further increase will cause a significant decrease in accuracy. Based on the abovementioned results, the learning rate is set to 0.005 in the following experiments.
(3) Number of heads in transformer block: The number of heads in the TE block is varied, with the head cardinality set to 2, 4, 8, and 16. Generally, an appropriate increase in the number of SA heads should enable the model to learn richer and more robust features. As the number of SA heads increases, the classification accuracy increases, but this increase comes at the cost of an increase in total network parameters, which can make network training more difficult and ultimately reduce its classification accuracy. Figure 2c shows that when the number of SA heads is equal to 4, the classification accuracy reaches the maximum value.
(4) Number of 3D convolution kernels: The influences of the numbers of 3D convolution kernels on the accuracy are illustrated in Figure 2d. The results show that the classification increased first and then decreased with more 3D kernels, and it peaks at 16 3D kernels. Overall, Figure 2d suggests that the classification accuracy is not significantly affected by the number of convolution kernels, indicating the stability of the model’s performance. Among them, the model with 16 kernels achieved the best performance.

According to the above hyperparameter experiments, the associated network architecture is properly determined. Finally, the parameters of optimal network architecture are listed in Table 1.

### 2.3. Ablation Experiments

To fully demonstrate the effectiveness of the proposed method, an analysis of the impact of different components on the overall model was conducted, focusing on their contribution to classification accuracy. Specifically, the whole model was divided into four components—SeAM, SaAM, 3D convolution module, and TE module. In total, eight combinations were considered, and the experimental results are summarized in Table 2.

First, for Case 1, the 3D convolution module yielded the lowest classification accuracy, but still a reasonable performance (96.09%). These results demonstrate that the 3D convolution kernel operation could learn the spectral–spatial joint information of the HSI data cube effectively. In comparison with the TE module, Case 2 exhibited better accuracy (96.29%). The improvement obtained illustrated that the transformer could extract spectral–spatial features of data more efficiently. The reason is that in comparison with limited receptive fields in convolution, the transformer has the capacity to model long-term dependencies. Case 3, which combined both 3D convolution and TE modules, obtained an accuracy of 97.78%—a more satisfying result than for Case 2. This result indicates that overall, the transformer has better advantages. It also implies that there is a long-range correlation between hyperspectral bands. With these three cases, the effectiveness of jointly extracting spatial and spectral information from raw 3D HSI data is demonstrated.

Second, regarding the attention module, for Case 4 and Case 5, both the SeAM and SaAM modules contribute to performance improvement. This confirms the effectiveness of attention mechanisms and also indicates that these two modules provide complementary information. In particular, the SeAM module experiences a larger performance gain (98.44% vs. 98.16%), indicating that spectral information is more useful in identifying differences. For Cases 6 and 7, when both attention mechanisms exist, the TE module again has a great influence on the classification.

Building on the previous analysis, it can be concluded that all proposed modules affect the classification performance to some extent, and among them, the spectral attention and transformer modules are more important. Overall, combining all modules exhibits the best recognition results.

### 2.4. Comparative Performance of Various Methods

In pursuit of a more comprehensive evaluation of the efficacy of the presented model, this subsection investigates representative models commonly utilized in hyperspectral imaging scenarios on both original full-spectrum and hyperspectral images to classify turtle shells of varying growth years.

#### 2.4.1. Discrimination Results of Representative Models Using Only Spectral Information

Four representative models (SVM, PLS–DA, linear discriminant analysis (LDA), and the 1DCNN deep learning model) using only spectral information associated with classic wavelength selection algorithms (SPA [31], uninformative variable elimination (UVE) [32], and CARS [33]) were established and investigated. Regarding the SVM, the linear kernel function was employed for the purpose of classification. A grid search procedure was utilized to ascertain the optimal values of the regularization parameter and kernel function parameter that yielded the highest accuracy. For PLS–DA, training was performed with different component counts, followed by cross-validation to evaluate their respective performances and determine the optimal performance. LDA utilized default parameters, while the 1DCNN comprised three convolutional layers interleaved with two max-pooling layers and batch normalization, followed by three fully connected layers. The quantitative metric comparisons of different methods, including precision, specificity, sensitivity, and accuracy, can be found in Table 3.

For full-spectra models, as shown in Table 3, the classification results of each model were all acceptable, with an accuracy higher than 90%. The SVM model had the worst performance with 92.62% accuracy, while the LDA model and PLS–DA model obtained close and better results with 94.56% accuracy. In comparison, 1DCNN showed slightly more precise prediction than PLS–DA and LDA models, with an accuracy of 94.73%. Thus, the results illustrated the feasibility of using only spectral information in the hyperspectral imaging data for the growth-year identification of turtle shells. Also, it further verified that hyperspectral imaging is able to capture growth year-related differences in the chemical compositions of turtle shells.

Regarding wavelength selection, it was observed that the overall classification accuracy employing the selected optimal band was inferior to the corresponding full-spectrum models. These phenomena were also observed in some studies using hyperspectral imaging, such as wavelength selection algorithms employed for the discrimination of *Chrysanthemum* varieties [34]. This might be due to the fact that, as the number of wavelengths was reduced by more than 85% after optimal wavelength selection, it may eliminate some useful information relating to the small differences in the chemical properties of turtle shells. Moreover, it was noticed that different feature-band selection algorithms also have a great impact on subsequent classification performance, especially for the traditional machine learning methods: SVM, PLS–DA, and LDA. Among the characteristic wavelength selection algorithms, CARS performed the best, still with a decrease of 1–3% compared to that using full spectral information. Consistent with the results based on full wavelengths, the 1DCNN model still obtained the best result with an accuracy of 93.16%, which was also relatively insensitive to various types of wavelength selection algorithms. This again proved that the deep spectral features learned by 1DCNN are more distinguishable.

In all, these results indicate that deep learning combined with only spectral information is effective and also has the advantage of being able to learn deep spectral features automatically, compared to traditional machine learning algorithms. Even without any optimal wavelength extraction, 1DCNN based on full wavelengths could be a reasonably good model for the growth-year identification of turtle shells.

#### 2.4.2. Comparing with Representative Deep Learning-Based Methods

To further evaluate the performance, the proposed SSA–3DTE model was compared with five representative classical deep learning-based methods: 2DCNN [35], 3DCNN [36], hybrid spectral CNN (HybridSN) (2DCNN + 3DCNN) [37], residual network (ResNet18) [38], and SE–ResNet18 [39]. For each network architecture parameter, please refer to the corresponding literature. For a fair comparison, we used the same optimizer settings and loss functions, and the classification results are summarized in Table 4.

First, as shown, the 2DCNN model had the lowest performance, which is because the 2D convolution kernels are not suitable for 3D hyperspectral cube data. That is, 2D convolution convolved in spatial dimensions and then summed in the spectral dimension, which leads to spectral feature information loss. The unsatisfactory results also imply the importance of spectral information. However, we note that for the deeper ResNet18 network, it also obtains better performance. Despite the limitations of convolving receptive fields, stacking multiple layers also helps to learn good representation. Also, residual structure effectively solves related problems such as gradient dispersion and difficulty of training as the depth of the network increases. In contrast, the shallow 3DCNN (two layers) that jointly extracts the feature information of the spatial and spectral dimensions can also significantly improve the accuracy. The HybridSN model, which combined 2DCNN and 3DCNN, had an accuracy of 95.93%, proving that 3D convolution is more suitable considering the 3D nature of the HSI data. It is worth mentioning that, compared with the accuracy of 3DCNN, the performance gain obtained by HybridSN is not significant.

Second, in comparison to ResNet18, the SE–ResNet18 model’s accuracy was further enhanced to 96.52% with the addition of the squeeze-and-excitation module. With the help of attention modules, the network pays different attention to different activation mappings, thus leading to a more discriminant feature learning and enhancing performance. This result also confirms the importance and effectiveness of the attention mechanism. Nevertheless, SE–ResNet18 (2DCNN model + attention) managed to achieve relatively good performance, but compared to our proposed model, these evaluation indicators were still around 2.4% lower. This illustrates that there exists a performance bottleneck in extracting features relying solely on two-dimensional convolution.

Third, considering the multi-band nature and the data redundancy of the hyperspectral data, 3DCNN also suffers from limitations in fully utilizing the spatial and spectral information. In contrast, the proposed model leverages the spatial–spectrum attention module to redistribute the weights of spatial and spectral information, leading to more powerful classification results. In conclusion, the proposed model outperforms all other methods on all four evaluation metrics, demonstrating its superiority in achieving the best classification accuracy.

**Figure 3:**
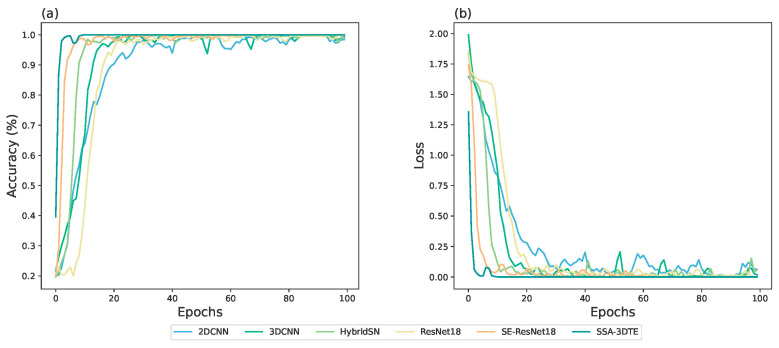
Accuracy curves (**a**) and loss curves (**b**) on the training sets of SSA–3DTE against the competitor models.

Figure 3 visualizes the overall accuracy and loss curves of the proposed model, SSA–3DTE, compared to other competitor models. It is evident that the SSA–3DTE model achieves the fastest convergence and the highest convergence accuracy. The accuracy of all models continuously improves as the number of training steps increases. Notably, 2DCNN shows the lowest initial accuracy and the slowest convergence speed, while SE–ResNet18 demonstrates high accuracy and the fastest convergence speed among the competitor models. The proposed model converges well in 20 epochs on the dataset. Overall, the accuracy and convergence of the curve are consistent with Table 4, and again the proposed SSA–3DTE model shows the best classification and robustness.

### 2.5. Confusion Matrix of Proposed Model

**Figure 4:**
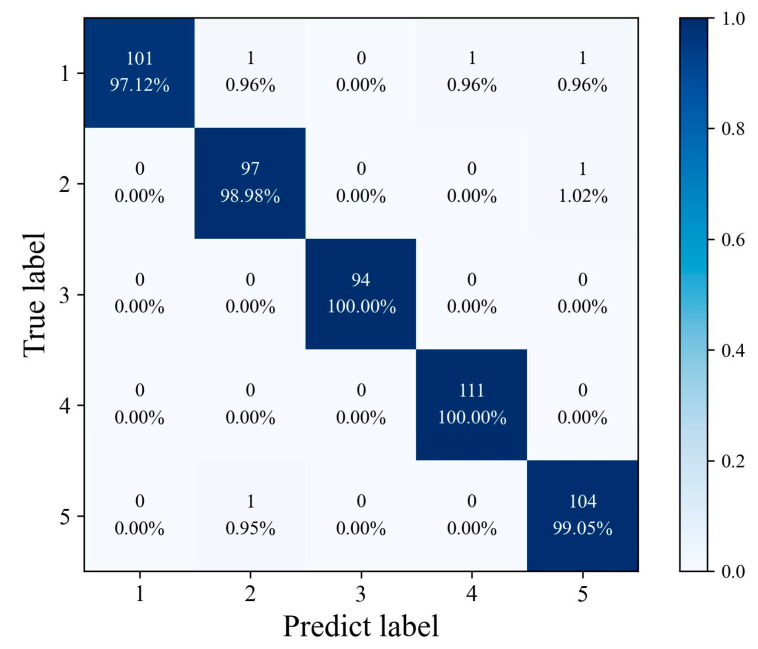
The confusion matrix of the SSA–3DTE model.

The classification confusion matrix of the SSA–3DTE model on the prediction set is shown in Figure 4. The diagonal of each matrix indicates correctly classified instances and true-positive rates, while the grid outside the diagonal represents incorrectly classified instances and false-negative rates. The confusion matrix offers a clear overview of the classification accuracy for each dataset category and allows for a better understanding of which categories are more likely to be misclassified by each measurement model. As can be seen from the figure, only a few samples are misclassified.

## 3. Materials and Preprocessing

### 3.1. Samples Preparation

The turtle shell samples were produced by the Shengchang turtle breeding farm in Jingshan City, Hubei Province, China. In total, 20 shell samples from turtles born in 2008, 2010, 2015, 2016, and 2017 were collected in 2021. Thus, the growth years of the turtle shell samples were 4, 5, 6, 11 and 13 years, respectively. These samples were authenticated by authoritative experts from the China Academy of Chinese Medical Sciences, and the results indicated that the samples possess uniform quality and variety.

### 3.2. Hyperspectral Imaging System and Image Acquisition

A laboratory-based push-broom HySpex series HSI spectrometer (Norsk Elektro Optikk AS, Oslo, Norway) was employed to obtain the hyperspectral image. The HSI spectrometer was made up of an N3124 SWIP lens (H-S16, Norsk Elektro Optikk, Oslo, Norway), a mobile platform, a computer with built-in software, and two 150 W tungsten halogen lamps used to illuminate the sample stage. The camera lens was set at a distance of 20–30 cm from the sample and collected images over a wavelength range of 948.72–2512.97 nm with 5.43 nm spectral resolution, for a total of 288 bands. Line-by-line images were acquired using a conveyor unit that covered the spatial shape of the samples and moved at a scanning speed of 1.5 mm/s. The original spectrometer employed in HSI features a spatial resolution of 2771 × 384 pixels (height × width). Hyperspectral image acquisition was performed for the front and back of each sample. All samples were securely placed onto the conveyor unit utilized for the acquisition of HSI data, ensuring that minimal bias was caused by surface tilt.

### 3.3. Hyperspectral Image Calibration

To mitigate the impact of uneven light-source intensity distribution and camera dark current noise on the acquired sample data, the raw intensity image was subjected to calibration using white and dark references. The white reference image was obtained by using a standard whiteboard with Lambertian features exhibiting nearly 100% reflectance, while the dark reference image was acquired by covering the lens with an opaque cap with nearly zero reflectance. The final corrected hyperspectral image, denoted as $R_{cal}$, can be automatically calculated by the following formula [40]:

$$R_{cal}=\frac{R_{raw}-R_{dark}}{R_{white}-R_{dark}},$$

where $R_{raw}$ is the raw hyperspectral image, $R_{dark}$ is the dark reference image, and $R_{white}$ is the white reference image. Subsequent image processing and analysis were performed on the $R_{cal}$.

### 3.4. ROI Selection and Dimension Reduction

The turtle shells, considered the regions of interest (ROIs), were segmented from the black background using ENVI 5.3 software, allowing for the extraction of all spectral and spatial information within each ROI. The extracted turtle shell samples displayed a uniform width of 384 pixels and manifested height discrepancies ranging from 610 to 810 pixels, attributable to the variations in morphological dimensions.

Each pixel in a hyperspectral image is associated with a continuous spectral curve that comprises tens to hundreds of narrow bands. However, the vast amount of data and redundant information lead to the curse of dimensionality, also known as the Hughes effect [41]. In addition, more data increase the computational complexity. As a powerful technique for dimensionality reduction, principal component analysis (PCA) is widely utilized to extract valuable features from hyperspectral images. Hence, before further model development, PCA was initially conducted to reduce redundant information by consolidating the majority of spectrum information.

## 4. Methods

**Figure 5:**
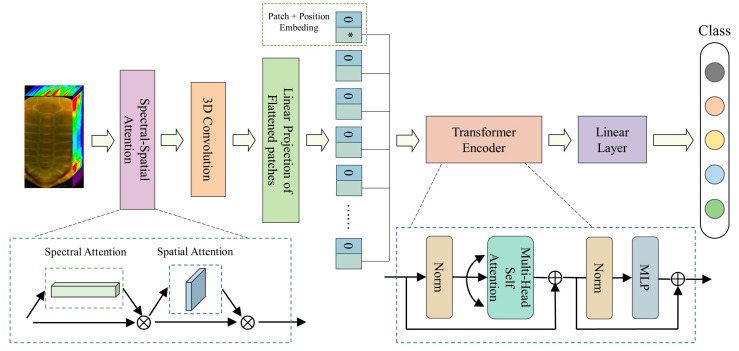
Flowchart of the SSA–3DTE model. * represents a learnable embedding vector.

This section presents the proposed SSA–3DTE model for the classification of turtle shell years. Figure 5 illustrates the overall architecture of the SSA–3DTE model, which is composed of three parts—the SSA block, the 3D convolution layers, and the TE module.

### 4.1. Spectral–Spatial Attention Block

The proposed model adopts the SSA module to adaptively recalibrate spectral bands and selectively refine spatial information, thereby enhancing the learned spectral–spatial features. This approach can effectively improve the feature extraction capability of the network.

Given a hyperspectral cube map $v\;\in \;R^{H\times W\times C}$, generating a 1D spectral attention map $M_{se}\;\in \;R^{1\times 1\times C}$ and a 2D spatial attention map $M_{sa}\;\in \;R^{H\times W\times 1}$, where C represents the spectral dimension, while H and W represent the spatial height and width, respectively. The overall SSA module computation can be summarized as

(1) $$v^{\prime }=M_{se}\left(v\right)\;⊗v\;V=M_{sa}\left(v^{\prime }\right)\;⊗v^{\prime }$$

where ⊗ denotes the elementwise multiplication. Details on the computation of the two attentions are provided below.

#### 4.1.1. Spectral Attention Module

To adaptively recalibrate the spectral information, the spectral attention module (SeAM) allocates varying weights to individual spectral channels. These weights are determined by their contribution to feature representation and classification, enabling the network to emphasize informative spectral bands and acquire more relevant features for enhanced performance. The SeAM is computed as:

(2) $$M_{se}^{\prime }\left(v\right)=W_{1}\left(\delta \left(W_{0}\left(v_{se}^{avg}\right)\right)\right)+W_{1}\left(\delta \left(W_{0}\left(v_{se}^{max}\right)\right)\right)\;M_{se}^{\prime }\left(v\right)=\sigma (M_{se}^{\prime }\left(v\right))$$

where $v_{se}^{avg}$ and $v_{se}^{max}$ are 1×1×C vectors produced by applying global average pooling and global max-pooling operations, respectively. The first fully connected (FC) layer, parameterized by $W_{0}$ with reduction ratio r, serves as a dimensionality-reduction layer, while the second is used to increase the dimensionality of date by parameter $W_{1}$. $W_{0}\;\in \;R^{\frac{C}{r}\times C}$, $W_{1}\;\in \;R^{C\times \frac{C}{r}}$, $M_{se}\;\in \;R^{C\times 1\times 1}$, $W_{0}$ and $W_{1}$ are shared weights. δ presents the ReLU activation function, δ denotes the sigmoid function. The SeAM generates the final output $v^{\prime }$ by multiplying the input v with the computed spectral weight vector $M_{se}$.

**Figure 6:**
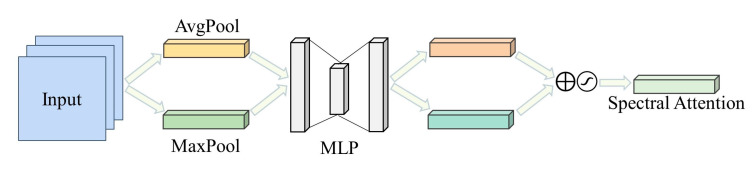
Spectral Attention Module.

The spectral attention module, depicted in Figure 6, leverages global average and global max pooling to extract the spectral features from the hyperspectral image. The resulting features are then processed by two FC layers with nonlinear activation functions to generate two pooling-channel representations. The module further correlates the two channels to derive spectral weights that emphasize informative features and attenuate noisy ones. Finally, the spectral weights are applied to the input feature map via element-wise multiplication, enabling the extraction of more discriminative and informative features.

#### 4.1.2. Spatial Attention Module

The spatial attention module (SaAM) module leverages the inherent inter-spatial relationships of features to identify informative regions in the hyperspectral image, highlighting the “where” aspect of feature learning. The spatial attention map calculation process is formulated as

(3) $$M_{sa}^{\prime }\left(v^{\prime }\right)=\left[v_{sa}^{avg},\;v_{sa}^{max}\right]*\;W_{2}\;M_{sa}^{\prime }\left(v^{\prime }\right)=\sigma (M_{sa}^{\prime }\left(v^{\prime }\right))$$

where $v_{sa}^{avg}$, $v_{sa}^{max}\;\in \;R^{H\times W\times 1}$, each represents average-pooled features and max-pooled features across the channel. ∗ is the convolution operation with a 7×7 filter. These two features are concatenated horizontally and fed as input to a new convolutional layer, which is activated by a sigmoid function. Finally, the spatial attention map $M_{sa}$ is multiplied with the input feature spectrawise to produce the final output V.

**Figure 7:**
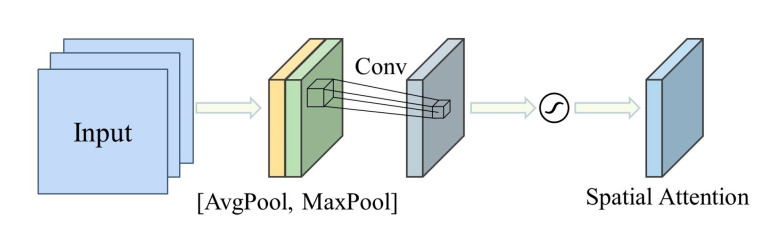
Spatial Attention Module.

The spatial attention module, as illustrated in Figure 7, first concatenates the features generated by global average pooling and max pooling along the channel axis to generate an efficient feature descriptor. This approach is effective in highlighting informative regions [42]. Next, the concatenated feature descriptor is convolved to capture the inner-spatial relationship between features, indicating where to emphasize or suppress. Finally, the spatial-wise input is multiplied element-wise with the spatial feature map to obtain the output feature map.

### 4.2. 3D Convolution Block

The output of the SSA mechanism maintains the 3D cube format, which presents an opportunity to leverage 3D spatial filtering for efficiently extracting both spectral and spatial features simultaneously [43]. In this regard, 3D convolution is employed to extract spectral–spatial features of each sample patch. Specifically, the jth feature cube at position (x, y, z) in the ith layer is calculated by:

(4) $$V_{ij}^{xyz}=\Phi \left(\sum_{m}\sum_{h=0}^{H_{i}-1}\sum_{w=0}^{W_{i}-1}\sum_{r=0}^{R_{i}-1}\omega_{ijk}^{hwr}V_{\left(i-1\right)k}^{\left(x+h)(y+w)(z+r\right)}+b_{ij}\right)$$

where m denotes the feature map in layer i−1 that is connected to the current jth feature map. The height and width of the spatial convolution kernel are represented by $H_{i}$ and $W_{i}$, respectively. $R_{i}$ is the depth of the convolution kernel in spectral dimension, $\omega_{ijk}^{hwr}$ is the weight at position (h, w, r) connected to the mth feature map, and $b_{ij}$ is the bias and $\Phi \left(\cdot \right)$ is the activation function.

This module utilizes two convolution layers, each with a 3×3×3 convolution kernel, no pooling, stride 1, and the same padding operation. Batch normalization and an ReLU activation function follow each convolution layer.

### 4.3. Transformer Encoder Block

Despite its advantage, the CNN model has limitations due to its kernel size and number of layers, which can prevent them from capturing the long-range dependencies in the input data and potentially ignoring some important sequence information. To overcome these constraints, a TE block is employed, which offers a self-attention (SA) variant to further extract image features [30].

Since the encoder model of the transformer is used instead of the decoder model, the position information of each vector is incorporated by transforming the sequence into a linear embedding sequence. This process involves segmenting the input data, which are the feature maps generated by the 3D convolution block, into patches and flattening them into a sequence of vectors. Additionally, a learnable embedding vector $V_{class}$ is added for classification purposes, followed by the addition of position encoding $E_{pos}$ for each vector. The process is represented as

(5) $$V_{in}=\left[V_{class};\;V_{p}^{1}E;\;V_{p}^{1}E;\ldots ;V_{p}^{N}E\right]+E_{pos}$$

where E represents the linear transformation layer.

**Figure 8:**
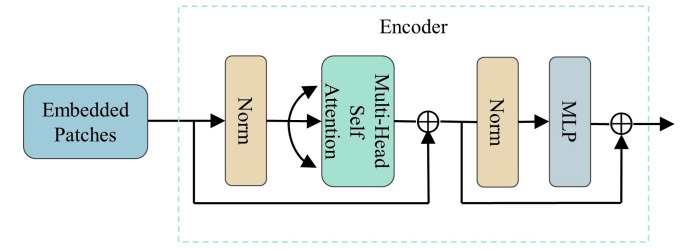
Multi-Head Self-Attention Module.

The encoder block consists of several components, including two normalization layers (LN), an MSA, and a multilayer perceptron (MLP) layer. The residual connection is implemented before MSA and MLP, and the specific structure is shown in Figure 8. The MSA mechanism, which is based on the SA mechanism, captures correlations among feature sequences effectively. Three learnable weight matrices, $W_{Q}$, $W_{K}$, and $W_{V}$, are defined in advance to linearly map transform the input matrix into three 3D invariant matrices—queries Q, keys K, and values V. In summary, the SA is formulated as follows:

(6) $$SA=Attention\left(Q,\;K,\;V\right)=softmax\left(\frac{QK^{T}}{\sqrt{d_{K}}}\right)V$$

where $d_{K}$ is the dimension of K.

In order to better capture the interdependencies among feature sequences, multiple SA mechanisms are used to calculate multiple head-attention values. The results from each head attention are then concatenated together. This process can be mathematically represented as

(7) $$MSA\left(Q,\;K,\;V\right)=Concat\left({SA}_{1},{SA}_{1},\ldots ,{SA}_{h}\;\right)W$$

where W is the weight matrix and h represents the number of attention heads.

Subsequently, the learned weight matrix from the previous step is fed into the MLP layer, which consists of two layers with a Gaussian error linear unit (GELU) activation function. This activation function incorporates the concept of random regularization, allowing the network to converge more efficiently and enhancing the model’s generalization capabilities [44].

### 4.4. Overview of the Proposed Model

The turtle shell sample data are obtained with shape (n, s, s, d), where n denotes the number of samples, d is the number of spectral bands after PCA dimension reduction, and s is the sample width and height, respectively. The sample data are first fed into the SSA module, which redistributes the weight of both spectral and spatial information. Notably, this process retains the original shape of the input feature map, resulting in an output sample data shape of (n, s, s, d). Following this, the 3D convolution layers are used to obtain feature cubes, each sized (m, m, k).

In the next step, each feature cube with shape (m, m, k) is segmented into an $\frac{m\times m}{p\times p}$ sequence of flattened 2D patches with shape (p, p, k), where p is set to 8. However, the transformer model expects a 2D matrix of size N×D as an input (removing the Batch_size dimension), where $N=\frac{m\times m}{p\times p}$ is the length of the sequence and D=p×p×k is each vector of the sequence dimension. Therefore, the $\frac{m\times m}{p\times p}$ 2D patch is reshaped into a matrix of shape $\left(\frac{m\times m}{p\times p},\;k\times p\times p\right)$ and undergoes linear transformation to create the 2D matrix with a shape of $\left(N,\;D\right)$. Afterward, the embedding vector $V_{class}$ and the position code $E_{pos}$ are introduced and a size matrix (Batch_size, N+1, D) is created (adding the Batch_size dimension), which serves as the input to the encoder block, and the encoder module continues extracting image features. Finally, the classification results are output through the linear layer and softmax function.

### 4.5. Experimental Settings

Evaluation Indicators: To analyze the proposed method, four quantitative indexes are employed, including accuracy, precision, recall, and $F_{1}score$, which are denoted as follows, respectively.

(8) $$Accuracy\left(\%\right)=\frac{TP+TN}{TP+FP+FN+TN}\times 100\;Precision\left(\%\right)=\frac{TP}{TP+FP}\times 100\;Recall\left(\%\right)=\frac{TP}{TP+FN}\times 100\;F_{1}score\left(\%\right)=\frac{2\times Precision\times Recall}{Precision+Recall}\times 100$$

where TP denotes true positives, TN is true negatives, FP represents false positives, and FN indicates false negatives. A higher value for each metric indicates better classification performance. For fairness, the training was repeated five times for each trial on the samples, and their average was taken as the final result.

Software and Experimental Configuration: The proposed model and the subsequent comparison methods were all implemented using the PyTorch environment with a Geforce RTX 3070 12-GB GPU server (NVIDIA, Santa Clara, CA, USA). The Adam optimizer was selected as the initial optimizer with a $\beta_{1}$ of 0.9 and a $\beta_{2}$ of 0.99, and the size of each minibatch was set to 32 for batch training. The initial weight matrix was obtained using Xavier standard normal distribution. The proposed DL model was trained for 100 epochs, using the cross-entropy function as the loss function.

Commonly, DL models require a significant amount of data for training to achieve better results, and yet the number of obtained turtle shell samples is rather limited. To alleviate the issue, each hyperspectral image of turtle shell was segmented without overlap into images of size 64×64. Finally, 2574 samples were obtained, of which the number of samples for five different years was 522, 492, 474, 552, and 534, respectively, and the samples were randomly split into training and test sets using an 8:2 ratio.

## 5. Conclusions

The identification of growth year is important for the quality inspection of turtle shells. In this study, for the first time, the feasibility of using the NIR hyperspectral model combined with a DL model to identify turtle-shell growth years was successfully investigated. To fully exploit the spatial and spectral information while reducing redundancy in hyperspectral data, three modules were developed. First, the SSA module was introduced to distinguish the different levels of importance of spectral and spatial information. Second, regarding the extracted 3D feature map, three-dimensional convolution and transformer modules were employed to effectively extract the local and global information for more efficient classification.

Our proposed model was compared to representative models (SVM, PLS–DA, LDA, and 1DCNN) using only spectral information and representative deep learning-based models (2DCNN, 3DCNN, HybridSN, ResNet18, and SE–ResNet18). Overall, using only spectral information, 1DCNN with full wavelengths obtained reasonable results (94.73%), outperforming conventional machine learning models (accuracies varied between 85.63 and 94.56%). This observation demonstrates its ability to learn deep spectral features automatically. In comparison, the average accuracy of the proposed model was as high as 98.94%, underscoring the necessity and effectiveness of incorporating spatial information. In addition, the ablation experiment also fully confirmed the important influence of each module on the final classification. In conclusion, this study introduces a novel approach by being the first to synergistically integrate HSI and DL techniques for the purpose of accurately determining the growth year of turtle shells. It may find practical applications in the herbal-medicine quality-control field, serving as an easy, efficient, and novel tool for turtle-shell growth-year identification.

## Figures and Tables

**Table 1:** The network configuration of SSA–3DTE architecture.

Layer (Type)	Kernel	Stride	Padding	Input Size	Output Size
SeAM	1×1	1	-	60×64×64	60×64×64
SaAM	7×7	1	3	60×64×64	60×64×64
Rearrange	-	-	-	60×64×64	1×60×64×64
Conv3d-1	3×3×3	1	same	1×60×64×64	16×60×64×64
BN1 + ReLU	-	-	-	16×60×64×64	16×60×64×64
Conv3d-2	3×3×3	1	same	16×60×64×64	16×60×64×64
BN2 + ReLU	-	-	-	16×60×64×64	16×60×64×64
Linear Embedding	-	-	-	16×60×64×64	513×64
TE block	-	-	-	513×64	64
Linear	-	-	-	64	5

**Table 2:** Ablation analysis of the SSA–3DTE model.

Case	Component				Accuracy (%)
	SeAM	SaAM	3D Conv	TE	
1	✕	✕	✓	✕	96.09 ±1.01
2	✕	✕	✕	✓	96.29 ±1.04
3	✕	✕	✓	✓	97.78 ±0.64
4	✕	✓	✓	✓	98.16 ±0.47
5	✓	✕	✓	✓	98.44 ±0.24
6	✓	✓	✕	✓	97.07 ±0.69
7	✓	✓	✓	✕	96.75 ±0.88
8	✓	✓	✓	✓	98.94 ±0.29

**Table 3:** Classification results of representative models employing only spectrum information.

Model	Extraction Method	Number of Bands	Accuracy (%)	Precision (%)	Recall (%)	$F_{1}score$ (%)
SVM	None	288	92.62	92.61	92.71	92.66
	SPA	46	85.63	85.60	85.48	85.54
	UVE	48	85.83	85.98	85.91	85.94
	CARS	31	91.26	91.54	91.20	91.37
LDA	None	288	94.56	94.64	94.64	94.64
	SPA	46	86.64	87.60	86.84	87.22
	UVE	48	89.78	89.90	89.90	89.90
	CARS	31	91.75	92.57	91.69	92.13
PLS–DA	None	288	94.56	94.66	94.62	94.64
	SPA	46	87.23	87.53	87.13	87.33
	UVE	48	90.37	90.90	90.22	90.56
	CARS	31	92.53	92.59	92.61	92.60
1DCNN	None	288	94.73	94.89	94.76	94.82
	SPA	46	90.82	91.53	90.66	91.09
	UVE	48	92.92	93.25	92.74	92.99
	CARS	31	93.16	93.56	93.00	93.28

**Table 4:** Comparison of classification performance with different classification models.

Class	2DCNN	3DCNN	HybridSN	ResNet18	SE–ResNet18	SSA–3DTE
1	91.15 ±3.15	93.08 ±1.58	95.57 ±1.09	95.39 ±2.84	95.38 ±2.08	97.89 ±1.58
2	91.43 ±1.85	96.33 ±1.16	96.94 ±1.25	95.31 ±3.02	95.51 ±1.85	98.98 ±0.72
3	88.94 ±4.23	92.13 ±3.41	93.19 ±1.21	94.26 ±3.33	94.26 ±2.45	98.72 ±1.75
4	97.84 ±1.02	98.20 ±1.42	98.38 ±1.17	98.38 ±0.75	99.82 ±0.41	99.82 ±0.40
5	93.14 ±1.83	96.57 ±2.29	95.23 ±2.78	94.67 ±1.86	97.14 ±1.65	99.24 ±0.89
Accuracy (%)	92.66 ±1.25	95.35 ±1.33	95.93 ±0.98	95.67 ±0.93	96.52 ±0.76	98.94 ±0.29
Precision (%)	92.79 ±1.23	95.39 ±1.28	95.99 ±1.03	95.72 ±0.87	96.56 ±0.79	98.92 ±0.31
Recall (%)	92.50 ±1.30	95.26 ±1.35	95.86 ±0.97	95.60 ±0.99	96.42 ±0.79	98.93 ±0.32
$F_{1}score$ (%)	92.64 ±1.27	95.32 ±1.32	95.92 ±1.00	95.66 ±0.93	96.49 ±0.79	98.93 ±0.31

## Data Availability

Data will be made available upon request.
